# Investigation of inter‐fraction target motion variations in the context of pencil beam scanned proton therapy in non‐small cell lung cancer patients

**DOI:** 10.1002/mp.14345

**Published:** 2020-07-09

**Authors:** Lydia A. den Otter, Renske M. Anakotta, Menkedina Weessies, Catharina T. G. Roos, Nanna M. Sijtsema, Christina T. Muijs, Margriet Dieters, Robin Wijsman, Esther G. C. Troost, Christian Richter, Arturs Meijers, Johannes A. Langendijk, Stefan Both, Antje‐Christin Knopf

**Affiliations:** ^1^ Department of Radiation Oncology University Medical Center Groningen University of Groningen Groningen 9713 GZ The Netherlands; ^2^ OncoRay – National Center for Radiation Research in Oncology, Faculty of Medicine and University Hospital Carl Gustav Carus Technische Universität Dresden Helmholtz‐Zentrum Dresden Rossendorf Germany; ^3^ Helmholtz‐Zentrum Dresden – Rossendorf Institute of Radiooncology OncoRay Germany; ^4^ Department of Radiotherapy and Radiation Oncology Faculty of Medicine and University Hospital Carl Gustav Carus Technische Universität Dresden Dresden Germany; ^5^ Partner Site Dresden, and German Cancer Research Center (DKFZ) German Cancer Consortium (DKTK) Heidelberg Germany; ^6^ National Center for Tumor Diseases (NCT) Partner Site Dresden Dresden Germany

**Keywords:** free breathing, inter‐fractional motion monitoring, lung cancer, moving targets, pencil beam scanning proton therapy

## Abstract

**Purpose:**

For locally advanced‐stage non‐small cell lung cancer (NSCLC), inter‐fraction target motion variations during the whole time span of a fractionated treatment course are assessed in a large and representative patient cohort. The primary objective is to develop a suitable motion monitoring strategy for pencil beam scanning proton therapy (PBS‐PT) treatments of NSCLC patients during free breathing.

**Methods:**

Weekly 4D computed tomography (4DCT; 41 patients) and daily 4D cone beam computed tomography (4DCBCT; 10 of 41 patients) scans were analyzed for a fully fractionated treatment course. Gross tumor volumes (GTVs) were contoured and the 3D displacement vectors of the centroid positions were compared for all scans. Furthermore, motion amplitude variations in different lung segments were statistically analyzed. The dosimetric impact of target motion variations and target motion assessment was investigated in exemplary patient cases.

**Results:**

The median observed centroid motion was 3.4 mm (range: 0.2–12.4 mm) with an average variation of 2.2 mm (range: 0.1–8.8 mm). Ten of 32 patients (31.3%) with an initial motion <5 mm increased beyond a 5‐mm motion amplitude during the treatment course. Motion observed in the 4DCBCT scans deviated on average 1.5 mm (range: 0.0–6.0 mm) from the motion observed in the 4DCTs. Larger motion variations for one example patient compromised treatment plan robustness while no dosimetric influence was seen due to motion assessment biases in another example case.

**Conclusions:**

Target motion variations were investigated during the course of radiotherapy for NSCLC patients. Patients with initial GTV motion amplitudes of < 2 mm can be assumed to be stable in motion during the treatment course. For treatments of NSCLC patients who exhibit motion amplitudes of > 2 mm, 4DCBCT should be considered for motion monitoring due to substantial motion variations observed.

## 1 Introduction

Pencil beam scanning proton therapy (PBS‐PT) is a conformal radiotherapy technique for treating cancer. For the treatment of locally advanced‐stage non‐small cell lung cancer (NSCLC), this technique can limit radiation dose to organs at risk (OARs).^1^, ^2^, ^3^, ^4^ However, the high target dose conformity and the respective steep dose gradient achieved when using scanned proton therapy lead to increased sensitivity to (breathing) motion and even more to changes in the motion behavior. Additionally, the interference of the moving target and the time structure of the scanned proton beam delivery causes “interplay” effects that result in dose heterogeneities.^5^, ^6^, ^7^

To benefit from the advantages of PBS‐PT, strategies are needed for moving targets to establish treatments that are robust to these motion and interplay effects. In case of NSCLC, the effects of breathing motion can be mitigated using breath‐hold techniques, rescanning, gating, tracking, abdominal compression, or a combination of the aforementioned.^3^, ^6^, ^8^, ^9^, ^10^, ^11^, ^12^, ^13^, ^14^ Furthermore, 4D robust optimization has shown to increase the robustness of treatment plans against the effects of breathing motion.^15^, ^16^, ^17^ The commercially available 4D robust optimization method uses the minimax robust optimization approach and includes multiple phases of a 4DCT scan as additional images during the optimization process.^18^, ^19^ Ribeiro et al.^20^ developed a comprehensive 4D robustness evaluation method (4DREM) to effectively check the combined influence of different uncertainties on PBS‐PT of moving targets. Ultimately, a 4D adaptive workflow could be the solution to obtain highly conformal dose distributions not compromised by motion and other anatomical variations occurring throughout the course of fractionated treatment.^21^

Patient selection is a key element in the treatment of moving targets with PBS‐PT. Literature states that NSCLC patients with gross tumor volume (GTV) motion amplitudes less than 5 mm can be safely treated using rescanning only to diminish interplay effects.^5^, ^22^, ^23^ However, it is not known which patients will show substantial motion variations during the course of treatment, for example, in motion amplitude. These changes might be driven by target volume shrinkage, atelectasis, or the comfort of the patient. So far, only a limited number of studies have investigated these motion variations for locally advanced‐stage NSCLC patients. Redmond et al.^24^ evaluated the motion variation in stage III NSCLC patients for two repeated 4DCTs that were acquired during the treatment course. They did not find major changes in motion compared to the planning 4DCT. Britton et al.^25^ also investigated motion variability using five to ten repeated 4DCTs for a total of eight patients with locally advanced NSCLC and found an increase in motion variation with increasing treatment weeks (*P* = 0.049). This increase indicated the need for weekly 4DCT motion monitoring. However, in this limited cohort, only two patients exhibited less than 5 mm motion at baseline.

In the study presented here, target motion variations were primarily investigated during the whole time span of a fractionated treatment course in a larger, representative population of locally advanced‐stage NSCLC patients. The motion evaluation is based on repeated weekly 4DCTs as well as on daily 4DCBCTs. The main purpose of the study is to develop a suitable motion monitoring strategy for the treatment of NSCLC patients with PBS‐PT during free breathing.

## 2 Materials and methods

### 2.A. Patient data

This study presents a retrospective inter‐institutional analysis using three different datasets. All datasets included repeated 4DCT images that were acquired on a weekly basis. Additionally, one of the datasets included daily pretreatment 4DCBCT acquisitions for 10 patients. The first dataset (A) contained 21 (N)SCLC patients who gave informed consent. The patients were part of a prospective cohort pilot study (REACT, ClinicalTrials.gov Identifier NCT03024138) to evaluate the impact of inter‐ and intra‐fraction motion variations on photon and proton dose distributions. These patients had advanced disease (stage III–IV) and received radiotherapy with curative intent in combination with chemotherapy (all but one patient) between December 2016 and November 2017.

The second dataset (B) was shared following a research agreement with OncoRay and the University Proton Therapy Dresden (Faculty of Medicine and University Hospital Carl Gustav Carus, Technische Universität Dresden, (Dresden, Germany). The dataset included six stage III NSCLC patients participating in a single‐center randomized clinical trial.^26^ The included patients received passively scattered proton therapy.

The third dataset (C) of 14 patients originates from the publicly available The Cancer Imaging Archive (TCIA) 4D Lung database.^27^ This particular dataset was provided by the Virginia Common Wealth University and contained 14 patients with locally advanced NSCLC who had undergone weekly repeated 4DCT imaging.^28^ This dataset was collected between 2008 and 2012.^29^, ^30^, ^31^ Accordingly, our analysis is based on a total of 41 patients. More specific patient information can be found in Table I.

**Table I:** Patient and tumor characteristics.

Factor	No. of cases	Cases (%)
Gender		
Female	16	39
Male	25	61
Tumor location		
Left upper lobe	14	34
Right upper lobe	18	44
Right middle lobe	3	7
Left lower lobe	0	0
Right lower lobe	6	15
Gross tumor volume		
<25 cm^3^	16	39
25–150 cm^3^	18	44
150–500 cm^3^	7	17
No. of 4DCTs		
2‐4	5	12
5‐6	33	81
7‐8	3	7

### 2.B. Image acquisition

Multiple weekly 4DCTs (on average five scans) were acquired for all patients. A Definition AS Open 64‐RT Pro CT scanner (Siemens Medical Systems, Erlangen, Germany), a Siemens Definition AS Open CT scanner, and a Brilliance Big Bore CT scanner (Philips Medical Systems, Andover, MA) were used for acquisition of datasets A, B, and C, respectively. 4DCT scans were reconstructed with 10 breathing phases for datasets A and C (phase‐based binning), and with eight phases for dataset B (amplitude binning). To determine the respiratory phases or amplitude, an Anzai belt (Anzai Medical, Tokyo, Japan) was used for datasets A and B, and a respiratory optical surrogate signal for dataset C (Real‐time Position Management (RPM) system, Varian Medical Systems, Inc.). Images were reconstructed with either 2.0 mm (datasets A and B) or 3.0 mm (dataset C) slice thickness, 1.0 mm in‐plane resolution, and image size of 512 × 512 pixels.

Daily 4DCBCT images were acquired in addition to the weekly 4DCTs for 10 patients included in dataset A using the Elekta X‐ray Volume Imager (XVI) version 5.0.2 imaging system (Elekta, Stockholm, Sweden). Each scan was acquired in 4 min with 50 degree/min gantry speed at 120 kVp, 16 mA per frame, 10 ms per frame (total 1350 frames), field‐of‐view of 25 × 25 cm, and application of a bowtie filter. Reconstruction of the 4D images was performed with 0.93 mm in‐plane resolution, 2.0 mm slice thickness, and image size of 270 × 270 pixels.

### 2.C. Contour generation

All planning and repeat 4DCTs were imported into RayStation (RaySearch Laboratories, Stockholm, Sweden). The original GTVs of the primary tumor were deformably warped (ANACONDA algorithm^32^) after a rigid registration towards the 50% phases of the planning 4DCT and the repeated 4DCTs. A radiation oncologist resident (R.A.) subsequently checked the warped contours and manually corrected when necessary (e.g., because of tumor volume shrinkage). Next, the GTVs were deformably warped to the other remaining phases of the repeat 4DCTs. Those warped contours were also visually checked and manually corrected by a radiation oncologist (M.D.) when necessary.

### 2.D. Inter‐fraction motion amplitude 4DCTs

The GTV centroid positions were determined for all phases of the available 4DCT scans. The largest distance was calculated between centroid positions for the eight (dataset B) or 10 phases (datasets A and C). This was done for three directions: superior–inferior (SI), anterior–posterior (AP), and right–left (RL). Subsequently, the total 3D displacement vector length was calculated using the three calculated distances: $\surd \left(SI^{2}+AP^{2}+RL^{2}\right)$. The resulting GTV motion amplitudes were compared between the weekly 4DCTs for each individual patient. Additionally, target volume changes and their correlation to motion changes were evaluated. Finally, the 4DCT phases depicting the largest motion amplitude (in terms of extreme target centroid position) were determined for a subpopulation of patients. These patients had a GTV located in the lower or middle lobe (originating from datasets A and C), and thus showed more extended motion amplitudes.

### 2.E. Inter‐fraction motion amplitudes of daily 4DCBCTs versus weekly 4DCTs

**Fig. 1:**
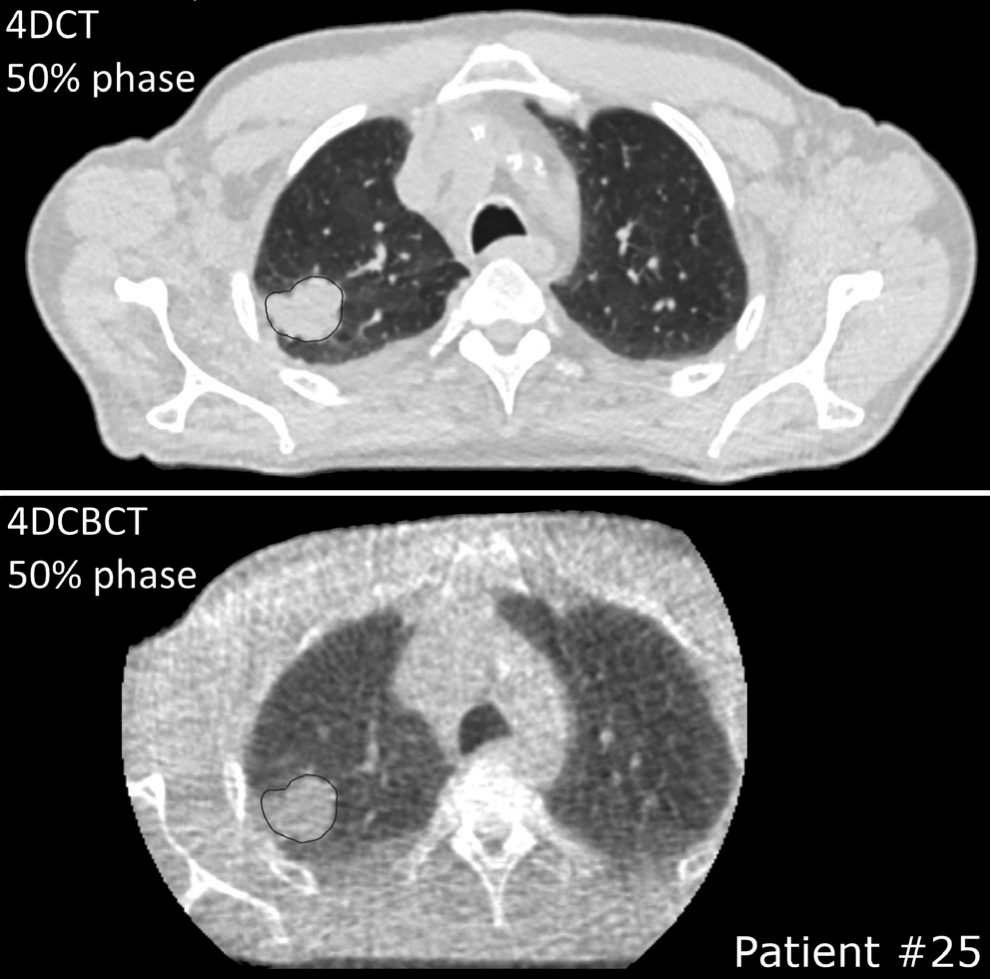
Example of a delineated GTV (transverse view) after deformable warping and correction on a 4DCT phase and the corresponding phase on a 4DCBCT scan.

For 10 patients originating from dataset A, daily 4DCBCTs in addition to the weekly 4DCTs were available. One patient was excluded because treatment was discontinued after 1 week. The same procedure for the motion amplitude evaluation was performed for the 4DCBCTs. The GTV contours were deformably warped from the nearest in time acquired 4DCT scan to the 4DCBCT scan, visually checked and manually corrected. This evaluation was performed for the 0% and 50% phases of the 4DCBCT scans only. A sample 4DCT and 4DCBCT from dataset A including the GTV contours are shown in Fig. 1. The GTV centroid motion of the 4DCBCTs was compared to the weekly 4DCTs in two ways: including either two 4DCT‐phases as well as the 10 phases of the 4DCT.

### 2.F. Location‐related differences in inter‐fraction motion amplitudes

The independent samples' Kruskall–Wallis test was performed to determine if the GTV motion found on the 4DCTs was different for different GTV locations (left upper lobe [LUL], right upper lobe [RUL], right middle lobe [RML], and the right lower lobe [RLL]). The population did not include patients with the GTV in the left lower lobe region. The GTV locations were compared for significant differences in motion amplitude. This was done pairwise to look into differences between upper and lower lobe as well as between left and right lobes. A value of *P* < 0.*050* was considered significant.

### 2.G. Dosimetric impact of target motion and target motion assessment

To illustrate possible dosimetric consequences of inter‐fraction motion changes, a plan robustness evaluation was performed in one example patient (#25), for whom the motion increased beyond 5.0 mm for two weekly 4DCTs. A clinical 3D robust‐optimized intensity‐modulated proton treatment (IMPT) plan was created. Treatment plan parameters included a prescribed dose in terms of relative biological effectiveness (RBE) of 60.0 Gy_RBE_ to the internal clinical target volume (iCTV) given in 25 fractions. A three‐beam configuration was chosen (two right posterior oblique, one right lateral). Within the iCTV, a density override to muscle tissue (1.050 g/cm^3^) was applied during optimization. Robustness was achieved using the minimax robustness approach, with 6.0‐mm setup uncertainties and 3% range uncertainties, using the average CT.^18^ These were the same robustness settings as applied in the clinic. The obtained 3D‐optimized plan was evaluated for its robustness on the average scans of the weekly repeat 4DCTs, applying a 2.0‐mm setup uncertainty and 3% range uncertainty as explained by Ribeiro et al.^20^ Robustness results were evaluated in terms of iCTV coverage and OAR doses through the voxel‐wise worst‐case doses: which is the computed minimum dose per voxel obtained from 14 scenario doses. OARs voxel‐wise worst‐case doses were computed as the maximum dose per voxel obtained from the 14 scenario doses. Nominal doses and voxel‐wise worst‐case doses were evaluated through dose volume histograms (DVHs).

Recently, 4D robust treatment plan optimization became available in RayStation. 4D robust optimization uses the minimax robust optimization approach and includes multiple phases of a 4DCT scan as additional images during the optimization process.^18^, ^19^ This way, differences in anatomy due to breathing motion are accounted for during optimization. As a compromise between computational time and a global robustness, it needs to be decided how many and which 4DCT phases should be included in the optimization process. To assess the dosimetric impact of employing the “wrong” extreme phases during 4D optimization, a 4D robust‐optimized treatment plan (6.0‐mm setup uncertainties and 3% range uncertainties) was created for an example patient (#37), based on the 0% and 50% phase (default extreme motion phases). The treatment plan was subsequently evaluated for its robustness on the actual extreme target motion phases of the 4DCT (60% and 90% phases; 6.0‐mm setup uncertainties and 3% range uncertainties).^20^

## 3 Results

### 3.A. Inter‐fraction motion amplitudes 4DCTs

**Fig. 2:**
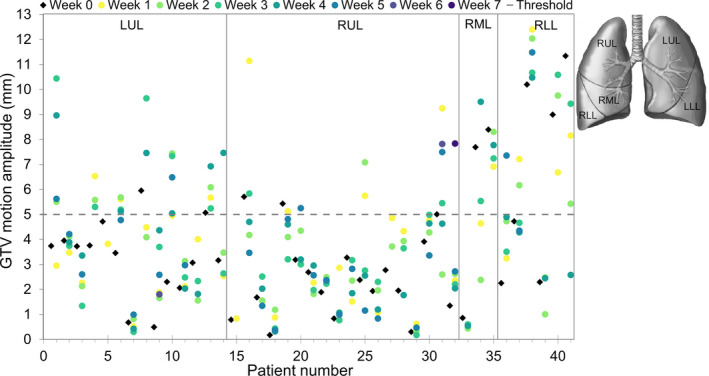
GTV centroid motion amplitudes for the 41 patients for the weekly 4DCTs. [Color figure can be viewed at wileyonlinelibrary.com]

**Fig. 3:**
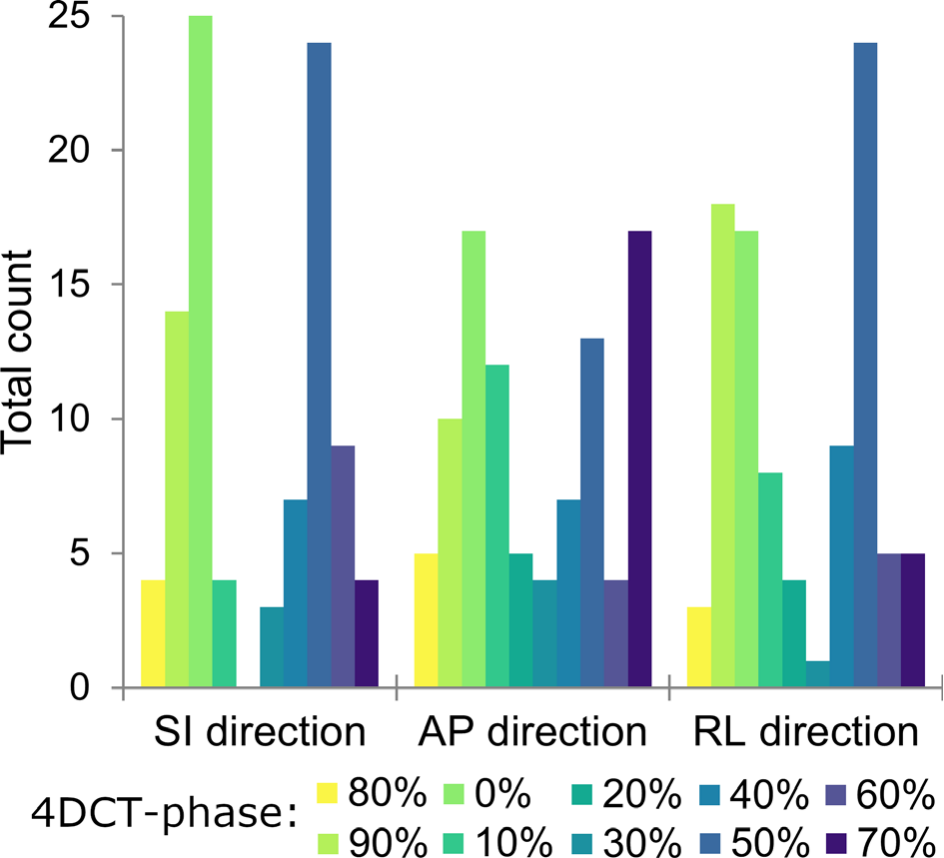
Total count that a specific 4DCT phase contained an extreme GTV centroid position for the middle/lower lobe targets (nine patients, a total of 47 4DCTs). [Color figure can be viewed at wileyonlinelibrary.com]

**Fig. 4:**
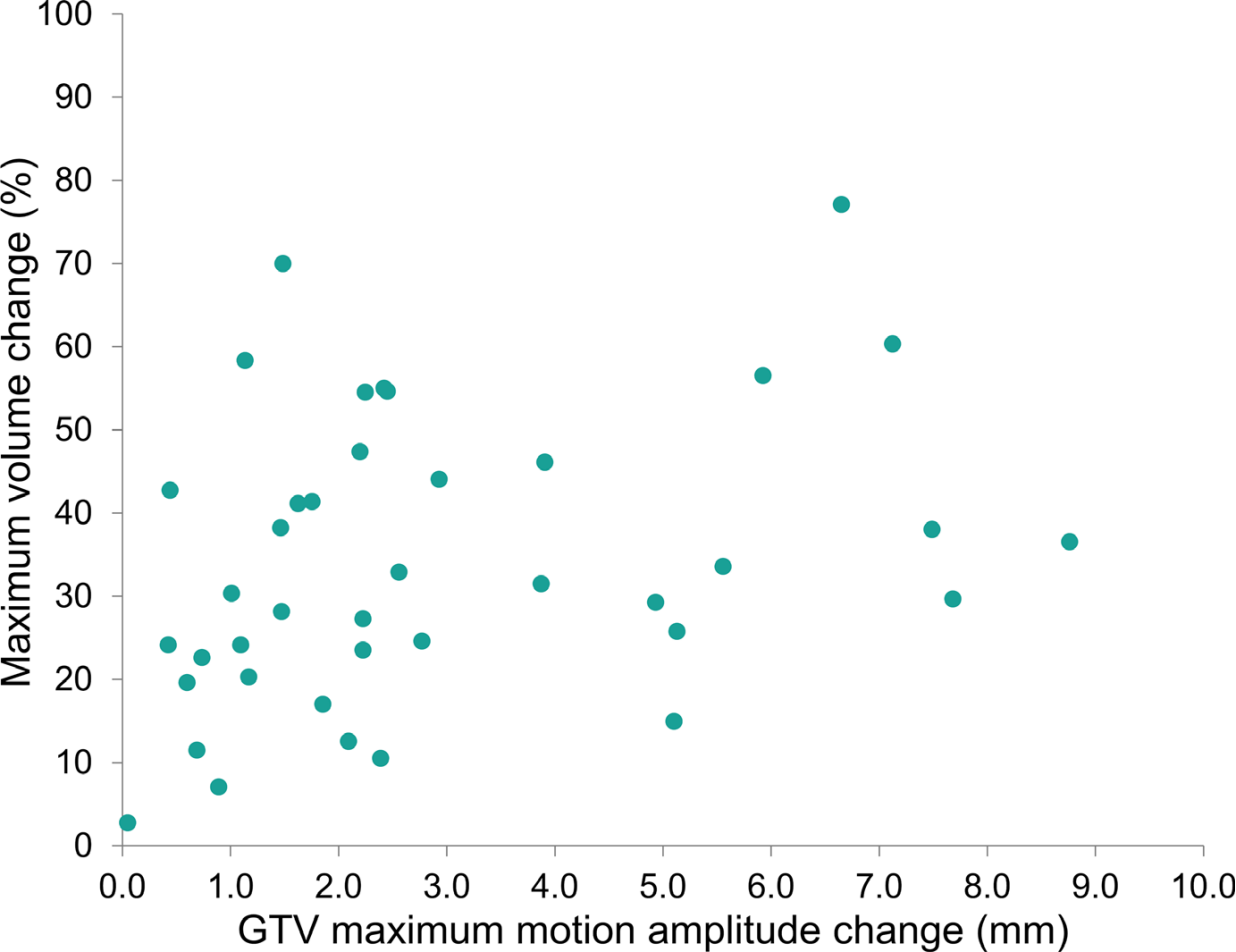
Maximum volume changes compared to GTV maximum motion amplitude changes. [Color figure can be viewed at wileyonlinelibrary.com]

The median observed amplitude of the GTV centroid motion was 3.4 mm over all time points (range: 0.2–12.4 mm; Fig. 2). The median variation in GTV motion amplitude for individual patients was 2.2 mm (range: 0.1–8.8 mm). GTV motion in week 0 revealed an initial motion of less than 5 mm for 32 out of 41 patients (78.0%). Of these patients, 10 of 32 (31.3%) showed GTV motion > 5 mm during the course of treatment for multiple weeks (median: 3 weeks, range: 1–4 weeks), extending up to 10.4‐mm amplitude motion for the most extreme case. Only patients with initial motion of less than 2 mm (12/41 patients) remained stable in motion (<5 mm) during the course of treatment. The extreme GTV motion amplitudes were captured in the 0% and 50% 4DCT phases for 52% (SI direction), 32% (AP direction), and 44% (RL direction) of the 4DCTs, see Fig. 3. The correlation between changes in target volume and GTV motion amplitude was examined using Spearman’s correlation coefficient. A significant (*P* = 0.012) correlation of 0.389 was found between the maximum volume change and maximum change in motion amplitude during the course of treatment **(**Fig. 4). The weekly measured target volumes are reported in the supplement (Fig. S1).

### 3.B. Inter‐fraction motion amplitudes of daily 4DCBCTs versus weekly 4DCTs

**Fig. 5:**
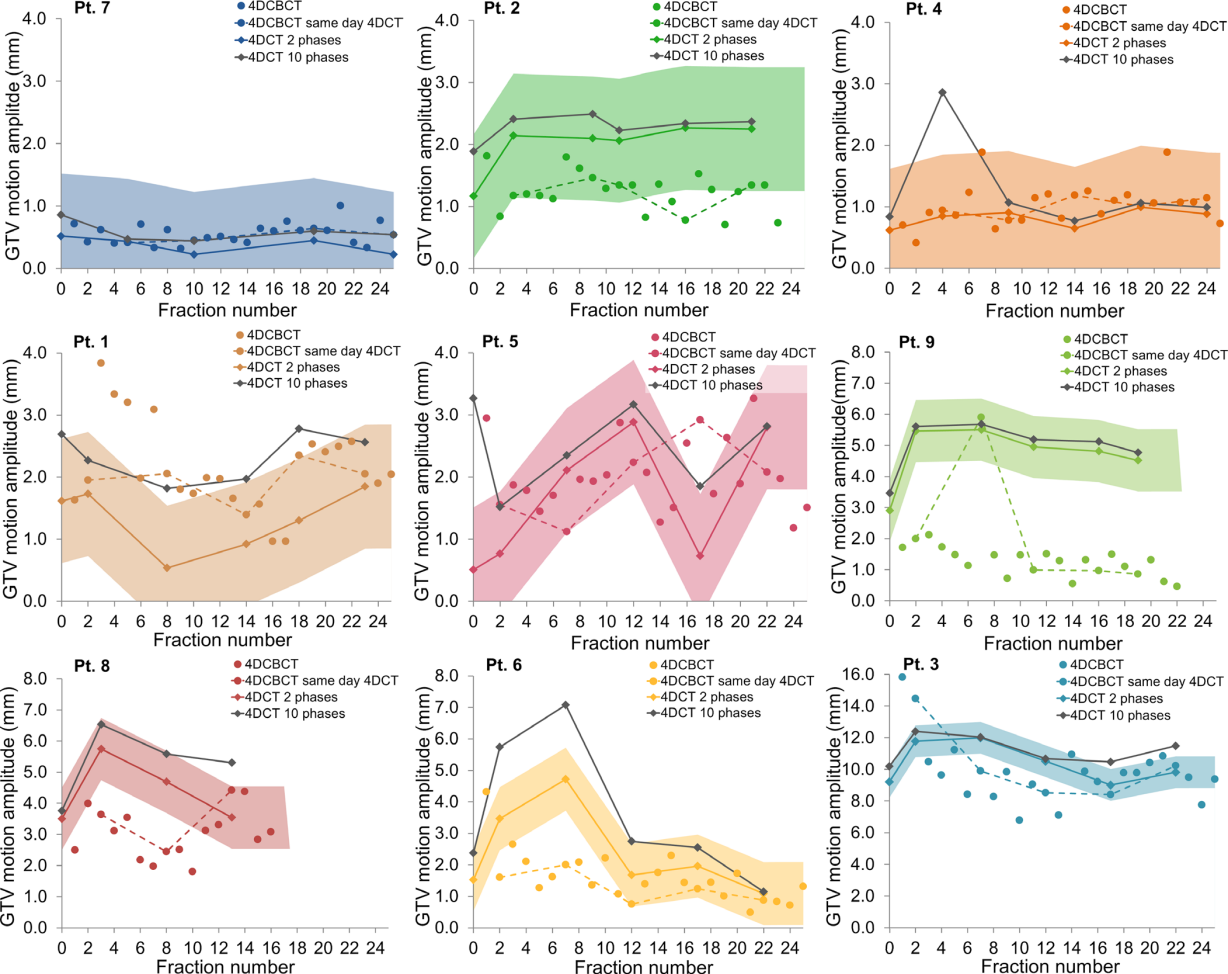
GTV centroid motion amplitudes from weekly 4DCTs together with the motion from daily 4DCBCTs. Patient data are presented in ascending order of motion amplitude. The highlighted colored areas show a margin of ± 1.0 mm. [Color figure can be viewed at wileyonlinelibrary.com]

The mean deviation between the centroid motion amplitudes of the weekly 4DCTs (all 10 phases) and daily 4DCBCTs was 1.5 mm (range: 0.0–6.0 mm; Fig. 5). When comparing the centroid motion of the 4DCBCT versus the motion amplitudes measured in the 4DCT for two phases, only two patients (4 and 7) followed the trend observed following the weekly 4DCT scans within a 1.0‐mm uncertainty band (Fig. 5). The other patients were in line with the 4DCT motion for only a part of the treatment course. One exception is patient #9, who showed smaller motion (±3 mm difference) in the 4DCBCT scans for all but one fraction.

### 3.C. Location‐related inter‐fraction motion amplitudes differences

To evaluate whether GTV motion depended on GTV location (i.e., lung lobe), pairwise comparisons were performed using the 4DCT information. Significant regional differences in motion were found (note that there were no left lower lobe patients in the study population). GTVs in the left upper lobe showed significantly larger motion compared to GTVs from the right upper lobe (mean motion 4.0 mm and 2.9 mm, respectively; *P* = 0.012). Furthermore, GTV motion in the right lower lobe (mean motion = 6.5 mm) was significantly larger than the motion found in the upper lobes: 2.9 mm (*P* = <0.001) and 4.0 mm (*P* = 0.020) in the right and left lobe, respectively.

### 3.D. Dosimetric impact of target motion and target motion assessment

**Fig. 6:**
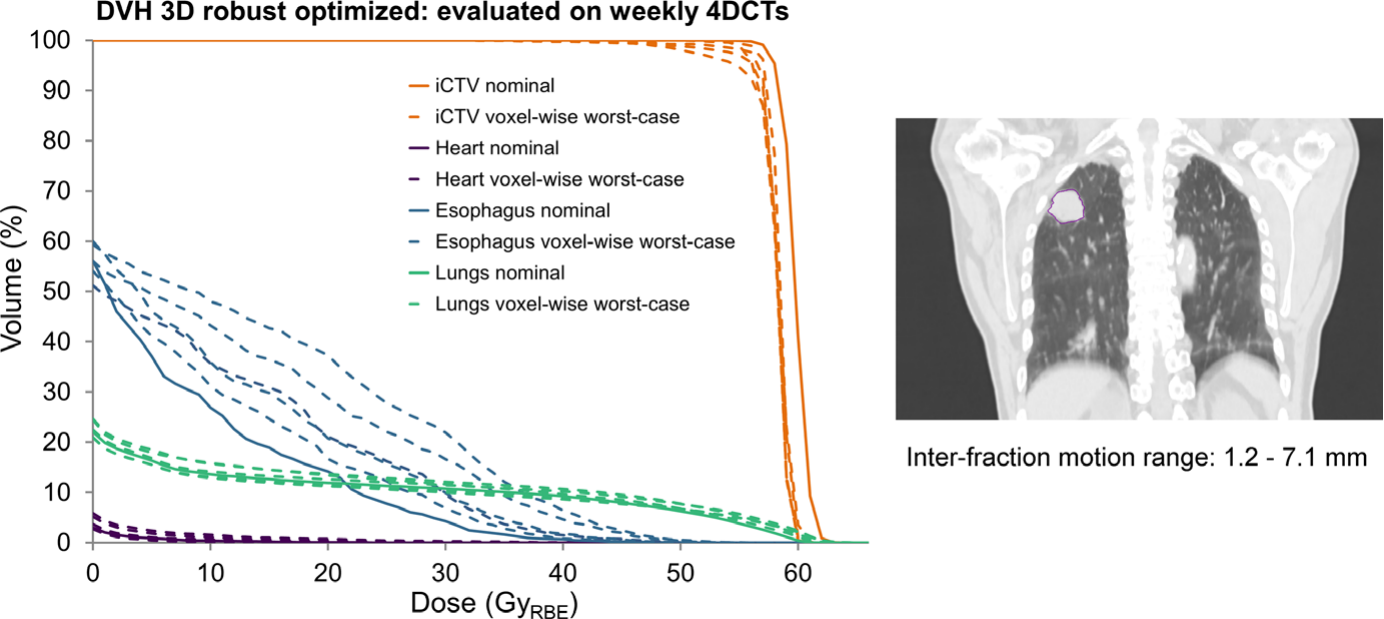
Dose volume histograms (DVHs) of the iCTV (orange), heart (purple), esophagus (blue), and lungs minus GTV (green) for a 3D robust‐optimized treatment plan. Depicted are the nominal doses (solid lines) and voxel‐wise worst‐case doses after robustness evaluation for the weekly 4DCTs (striped lines). A coronal view of the GTV location and the inter‐fraction motion range is shown on the right. [Color figure can be viewed at wileyonlinelibrary.com]

**Fig. 7:**
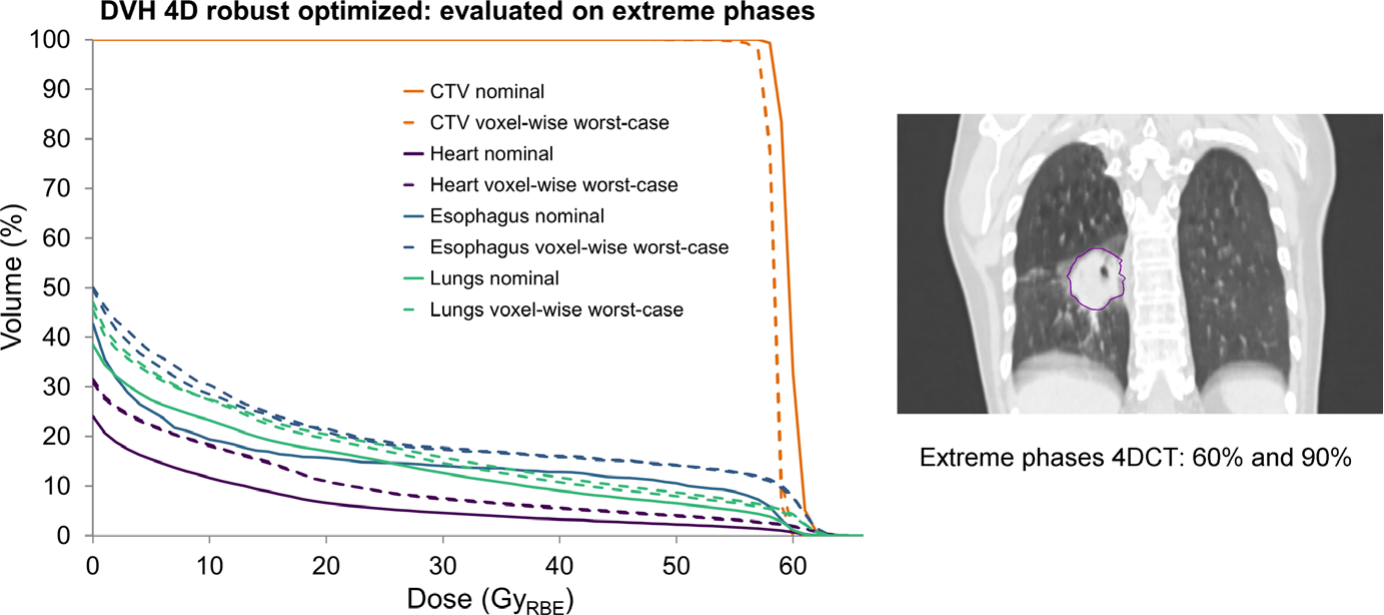
Dose volume histograms (DVHs) of the 4D robust‐optimized treatment plan using two phases (0% and 50%) for one sample patient. The nominal doses (solid lines) and voxel‐wise worst‐case doses on the actual extreme phases (60% and 90%) are shown (striped lines) for the CTV (orange), heart (purple), lungs minus GTV (green), and esophagus (blue). A coronal view of the GTV location is shown on the right. [Color figure can be viewed at wileyonlinelibrary.com]

The 3D‐optimized treatment plan for patient #25 was robustly evaluated through the voxel‐wise worst‐case doses. The iCTV coverage (D_98%_ [Gy_RBE_]) showed a deterioration of D_98%_ for all weeks (Fig. 6). This deterioration was the largest (51.12 Gy_RBE_ and 53.94 Gy_RBE_ D_98%_) for the repeated 4DCTs in the first 2 weeks, in which the measured GTV motion increased from 2.4 mm (week 0) to 5.7 mm and 7.1 mm, respectively. The dosimetric impact of considering the “wrong” extreme motion amplitude phases during 4D robust optimization is illustrated for an example case in Fig. 7. A small decrease in D_98%_ for the voxel‐wise worst‐case doses was observed for the CTV (D_98%_: 57.91 Gy_RBE_ [60% phase] and 57.96 Gy_RBE_ [90% phase]).

## 4 Discussion

In this study the main objective was to investigate GTV motion amplitude variations of locally advanced‐stage NSCLC patients during a fractionated treatment course. Approximately one third of the patients showed initially small GTV motion (<5 mm), which increased beyond 5 mm for one or multiple weeks during the course of treatment. Moreover, between individual patients, large differences were found in motion amplitude variations, ranging from “static” targets to “highly mobile” targets. Only GTVs that moved less than 2 mm prior to treatment remained at a stable low motion amplitude below 5 mm during treatment. For the patients with a GTV moving more than 2 mm, 4D motion monitoring on a daily basis will give insight in the patient‐specific motion trend and can therefore prevent nonrobust treatments by adapting treatments when necessary. However, further research is required to explore the (dosimetric) implications of the observed motion amplitude variations on scanned proton treatments.

Another finding was that the anatomical location of the GTV (i.e., the different lung lobes) influenced the motion extent. These findings are in line with those reported by Seppenwoolde et al.,^33^ Redmond et al.,^24^ and Britton et al.,^25^ who found significantly larger motion in the lower lung lobes. Motion management for patients with lower lobe GTVs might therefore be more challenging due to the generally found larger motion amplitudes.

We found a substantial volume reduction of the GTV during the course of radiotherapy (Fig. S1). Large volume reductions were also reported by Britton et al.,^25^ with 41.7% and 37.1% median tumor volume reduction for end‐inhalation and end‐exhalation phases, respectively. Brink et al.^34^ reported up till 60% decrease of volume and Erridge et al.^35^ reported at least 20% reduction of GTV volume for 40% of the patients. Notable volume and motion amplitude changes in our population were already observed halfway treatment. Patients with these early changes may benefit from adaptive treatment approaches.

Interestingly, in our population a significant correlation was found between maximum volume changes and motion variations, indicating that larger volume reductions can lead to increased motion amplitudes (Fig. 4). Future research could investigate more thoroughly in what way volume reductions and tumor motion amplitude variations correlate and influence the target coverage independently during proton treatments. This was unfortunately beyond the scope of this study.

Besides GTV volume reduction, other factors might also play an important role in motion changes, for example resolving atelectasis or pleural effusion. Patients’ comfort can change over the course of treatment and this can also influence breathing motion. Baseline drifts for example have been investigated by a number of studies for patients treated with stereotactic body radiation therapy (SBRT). Takao et al.^36^ examined 68 SBRT patients with real‐ time tumor‐tracking radiation therapy (RTRT) and fiducial markers. They found > 3 mm incidence of 3D vector‐calculated baseline drifts for 42.1% of the population after 10 min of treatment. Malinowski et al.^37^ found for 37 lung cancer patients that 13% showed > 5 mm baseline drift after 20 min of treatment. The incidence and severity of the baseline drift seem to increase with the duration of treatment. Moreover, Seppenwoolde et al.^33^ found motion changes between 1 and 5 mm due to hysteresis for 10 out of 21 tumors. It is important to consider these type of motion changes as well for scanned proton treatment and adjust treatment strategies accordingly.

GTV motion trends extracted from weekly repeated 4DCTs were not reflected in the extracted motion of the 4DCBCTs at corresponding days. The large deviation for patient 9 can be explained by the fact that this patient was highly claustrophobic and heavier breathing due to anxiety was noticed during the 4DCT imaging sessions. In general, as only the 0% and 50% 4DCBCT phases were evaluated, we expected a small systematic underestimation of motion as these phases do not necessarily encompass the extreme target position. However, also deviations between extracted motions amplitudes calculated with two phases of the 4DCBCT scans versus two phases of the 4DCT scans remained (Fig. 5). The 4DCBCT acquisition time was 4 min per scan, and baseline drifts could have taken place during the acquisition compared to the 4DCT acquisitions of 1 min. This could have influenced the resulting centroid motion amplitude comparison between the two modalities. However, Takao et al.^36^ found for their population that the incidence of baseline drifts rose after 3 min with only 8% of the population exhibiting drifts > 3 mm after 4 min of treatment. This was also measured during the actual treatment time, after patient setup and positioning verification. We therefore can only suspect a small influence of baseline drifts on the 4DCBCT acquisitions for a part of the patient population. With respect to the observed large day‐to‐day motion variations in the 4DCBCTs, we recommended base treatment adaptation decisions on observed trends as proposed by Meijers et al.^21^ rather than on single day recalculations.

We revealed that extreme GTV positions can occur in any 4DCT phase for NSCLC patients. In an exemplary evaluation, we did not find a substantial decrease in plan robustness when using other than the extreme motion phases for optimization. Still, our recommendation would be a careful phase selection to try capturing the full motion extent, whereas the 4D robust treatment planning time is kept reasonably limited.

In addition to currently published research, we investigated target motion variations using a comprehensive dataset that contains a substantially larger number of patients with four or more repeated 4DCTs as well as daily 4DCBCTs for a subset of patients. In contrast, previous studies analyzed only one or two repeat 4DCTs. Therefore, our study provides a deeper insight in occurring motion variations. Unfortunately, in our dataset, the GTVs are not evenly distributed within the lungs. There are notably more patients with GTVs located in the upper lobes. Nevertheless, significant differences in motion were found for the varying target locations.

Quality of the 4DCT and 4DCBCT images in this study may be an issue as some 4DCT scans contained binning artifacts. Optimization of the reconstruction process may improve the quality of the images and may therefore further optimize motion evaluation. For the reconstruction of the 4DCT and 4DCBCT images, two different signals for the binning methods were used, namely an external respiratory surrogate signal using the Anzai belt (4DCT) and the internal surrogate signal using the Amsterdam Shroud method by Zijp et al.^38^ (4DCBCT). The external and internal respiratory surrogate signals for image phase binning could influence the resulting image quality in a different way. Shieh et al.^39^ investigated and found negligible differences in image‐quality values for reconstructed 4DCBCTs between an external and internal respiratory signal surrogate. It was advised to verify these findings in a larger patient cohort. Nevertheless, we carefully conclude from these findings that differences in tumor motion estimation between 4DCT and 4DCBCT would be more apparent because of image‐quality differences between the two modalities than due to inaccuracy differences between the used respiratory signals for phase binning.

There exist differences in imaging accuracy between several brands of 4DCT scanners, which can result in errors during motion estimation. This was investigated by Hurkmans et al.^40^ They investigated the range of motion error for different types of CT scanners. The type of scanner used for datasets A and B of this study would be congruent with a range of motion error of 0.3 mm. For the third dataset, the CT scanner was of another brand and for those patients the error would be around 1.0 mm. We determined that patients would remain stable during treatment when a motion of less than 2 mm was measured before start of treatment. The inaccuracy in the value of 2 mm is < 1 mm.

Furthermore, the quality of the motion evaluation depended on the quality of delineations. We expect that even with a strict method of deformable warping of contours and manual checking afterward, uncertainties will remain. However, these uncertainties will be less than the inter‐observer variability for delineations.^41^ Major anatomical changes (e.g., GTV shrinkage) also make it difficult to propagate GTV contours using deformable image registration algorithms alone. A thorough checking with manual adjustments was performed in this study, to cope with this issue. Furthermore, it has been shown in other studies that it is possible to combine deformable image registration algorithms for contour propagation with other (semi)automatic techniques like a watershed‐cuts algorithm to improve the accuracy of the contours.^42^

## 5 Conclusions

Investigation of target motion variations during the course of radiotherapy for a large group of locally advanced‐stage NSCLC patients revealed that only initial GTV motion amplitudes of < 2 mm seem to remain stable during the treatment course. Patients fulfilling this criterion might be less challenging for PBS‐PT treatment during free breathing. For target motion amplitudes larger than 2 mm, we recommend 4DCBCT motion monitoring and base treatment adaptation decisions on observed trends.

## Conflict of Interest

All the co‐authors have no conflict of interest to disclose.

## Financial support

Financial support for this study was obtained from the University Medical Center Groningen: NCT03024138.

## Supporting information


**Figure S1**. Target volumes changes according to the weekly repeat 4DCTs for all patients. The volumes are shown for different lung regions as depicted in the schematic picture on the right.
