# Supplementary material for: Investigation of inter‐fraction target motion variations in the context of pencil beam scanned proton therapy in non‐small cell lung cancer patients
Source: Med Phys. 2020 Jul 9;47(9):3835–44. doi: 10.1002/mp.14345 (PMC7586844; doi:10.1002/mp.14345)
Supplement: Supplementary file 1 — Figure S1. Target volumes changes according to the weekly repeat 4DCTs for all patients. The volumes are shown for different lung regions as depicted in the schematic picture on the right. [file MP-47-3835-s001.doc]

**Supplementary materials**

**Target volumes changes**


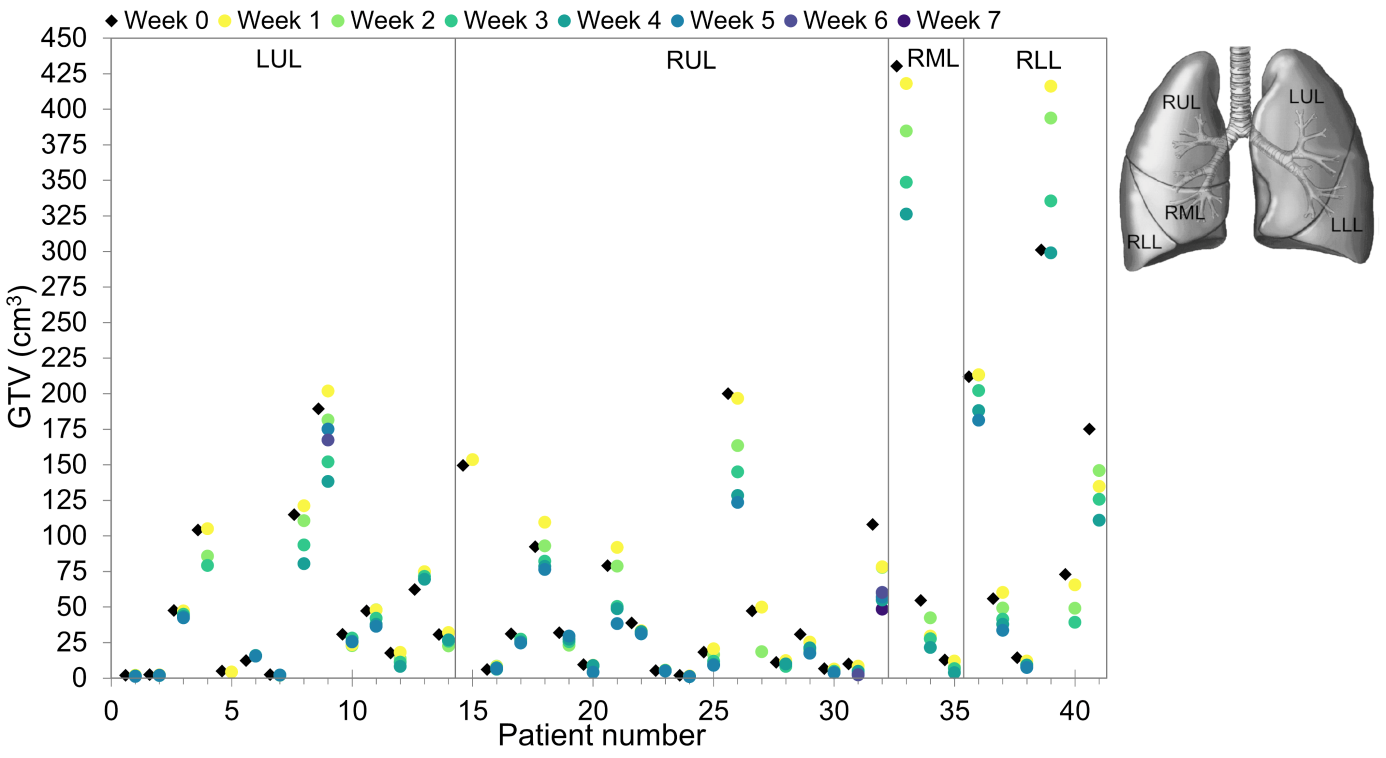
For each weekly repeat 4DCT the average volume of all phases was calculated and changes in GTV evaluated between the patients. Target volumes increased in week one to decrease again in the following weeks for 20 out of 41 patients **(Figure S-1)**. During treatment, the average GTV reduction (either from week 0 or from week 1) was 34.3% (standard deviation [SD]: 17.3%). Halfway through treatment this reduction was on average 24.5% (SD: 16.0%).

**Figure S-1**: Target volumes changes according to the weekly repeat 4DCTs for all patients. The volumes are shown for different lung regions as depicted in the schematic picture on the right.
